# Veränderungen und Entwicklungen in der präklinischen Notfallversorgung: Zentrale Herausforderungen für das Rettungsdienstmanagement

**DOI:** 10.1007/s00103-022-03588-x

**Published:** 2022-09-16

**Authors:** Daniel Lauer, Stephan Bandlow, Maik Rathje, Andreas Seidl, Harald Karutz

**Affiliations:** Fakultät Humanwissenschaften, MSH Medical School Hamburg, Am Kaiserkai 1, 20457 Hamburg, Deutschland

**Keywords:** Führung, Versorgungsstrategie, Demographischer Wandel, Digitalisierung, Gesundheitssystem, Leadership, Supply strategy, Demographic change, Digitalisation, Health care system

## Abstract

Der Rettungsdienst in Deutschland und das Management der gesamten präklinischen Notfallversorgung sehen sich aktuell mit zahlreichen Herausforderungen konfrontiert. Im letzten Jahrzehnt stiegen die Einsatzzahlen in nahezu allen Rettungsdienstbereichen kontinuierlich an, so dass die Vorhaltung von Rettungsmitteln vielerorts bereits deutlich verstärkt worden ist. Das Personalmanagement fällt jedoch zunehmend schwer und auch die technischen sowie medizinischen Anforderungen wachsen stetig. Zunehmend werden Stimmen laut, die eine grundlegende Reform der Notfallversorgung fordern.

Ziel dieses Beitrags ist es, einen Überblick über die aktuellen Entwicklungen, Trends und zukünftigen Herausforderungen zu geben, um die Anforderungen an das heutige und zukünftige Rettungsdienstmanagement deutlich zu machen. Verschiedene Themen und Handlungsfelder wurden dazu anhand einer nichtsystematischen Literaturrecherche herausgearbeitet. Die Anforderungen an das Rettungsdienstmanagement werden iterativ zusammengeführt.

Die Herausforderungen an den Rettungsdienst sind vielfältig und komplex, ebenso die Anforderungen an dessen Management. Die heterogene und kleingliedrige Organisationsstruktur des Rettungsdienstes im Bundesgebiet stellt eine wesentliche Herausforderung für Reformprozesse dar. Fehlende wissenschaftliche Kompetenzen im Rettungsdienst erschweren die Prozessoptimierung ebenfalls. Die zunehmende Akademisierung und Erforschung des Themenfeldes sind ausdrücklich zu begrüßen.

## Abstract (en)

Emergency medical services (EMS) and pre-hospital rescue management in Germany as a whole are currently confronted with numerous challenges. In the last decade, the number of calls has risen in almost every EMS area, resulting in a significant increase of ambulance provision. However, human resources management is becoming increasingly difficult and technical and medical requirements are rising steadily. Increasingly, voices are calling for a fundamental reform of emergency care.

The aim of this article is to give an overview of the current developments, trends and future challenges in order to illustrate the requirements for present and prospective rescue management. Different topics and areas of action were identified using a non-systematic literature search. The requirements for rescue management are brought together iteratively.

The challenges facing EMS are versatile and complex, as are the demands on its management. The heterogeneous and small-scale organisational structure of emergency medical services in Germany poses a major challenge for reform processes. The lack of scientific competencies in EMS also complicates process optimisation. The increasing academisation and research in this field are to be welcomed.

## Einleitung

Das Gesundheitswesen in Deutschland unterliegt insgesamt einem dynamischen Veränderungsprozess, der auch die Organisation und Struktur der präklinischen Notfallversorgung nicht unbeeinflusst lässt. In der letzten Dekade hat sich die Struktur des Berufsfeldes Rettungsdienst verändert. Sich wandelnde Rahmenbedingungen rettungsdienstlichen Handelns werden nachfolgend in einem orientierenden Gesamtüberblick dargestellt. Davon ausgehend werden Auswirkungen auf den Rettungsdienst skizziert und Konsequenzen für das zukünftige Rettungsdienstmanagement abgeleitet.

## Aktuelle Entwicklungen und Rahmenbedingungen

**Figure 1:**
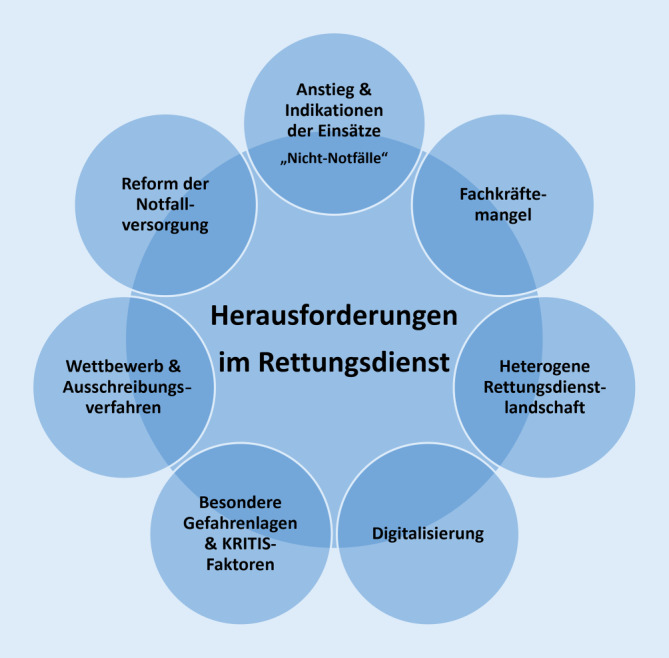

Über die letzten 20 Jahre war ein kontinuierlicher Anstieg der rettungsdienstlichen Einsatzzahlen zu verzeichnen. Jüngere Analysen zeigen jährliche Steigerungsraten von 4 % [1], lokal sind vereinzelt auch höhere Raten zu beobachten. Um das Mehr an Einsätzen gemäß den jeweils landesrechtlichen Vorgaben bedienen zu können, sind die Vorhaltungen in Form von zusätzlichen Rettungsfahrzeugen kontinuierlich erhöht worden. Auch der Personalkörper ist deutlich gewachsen; in den letzten 10 Jahren hat sich die Anzahl der sozialversicherungspflichtig Beschäftigten im Rettungsdienst nahezu verdoppelt [2]. In den letzten beiden Jahren bestimmte die COVID-19-Pandemie vielerorts den Alltag und stellt weiterhin das gesamte Gesundheitssystem vor besondere Herausforderungen. Vor und während der Pandemie wurden mehrere Ansätze zur Reform der Notfallversorgung vorgestellt, die aber bisher nicht zu einer tragfähigen Lösung konsolidiert wurden. Im Folgenden werden die relevanten Rahmenbedingungen und wichtige Entwicklungen der letzten Jahre dargestellt (Abb. 1).

### Sachstand Notfallsanitäter (NotSan)

Prof. Ahnefeld forderte bereits in den 1970er-Jahren eine dreijährige Berufsausbildung für Sanitäter auf gesetzlicher Grundlage [3]. Nach über 40 Jahren ist mit Inkrafttreten des Notfallsanitätergesetzes (NotSanG) am 01.01.2014 ein neues Berufsbild geschaffen worden, wodurch primär die notfallmedizinische Versorgungsqualität der Bevölkerung in Deutschland verbessert werden sollte. Notfallsanitätern ist ein höheres Maß an erweiterten fachlichen Kompetenzen bei der Versorgung erkrankter oder verletzter Menschen zugesprochen worden [4].

Das Berufsbild des Notfallsanitäters ist den Gesundheitsfachberufen zuzuordnen und stellt im bundesdeutschen Rettungsdienst die höchste nichtärztliche Qualifikation dar [5]. Um den gestiegenen Anforderungen im Aufgabenspektrum des Notfallsanitäters zu begegnen, wurden mit dem NotSanG und der dazugehörigen Ausbildungs- und Prüfungsverordnung (NotSan-APrV) der Aufgaben- und Kompetenzbereich sowie die inhaltliche Ausbildung erweitert. Mit der Einführung des § 2a NotSanG wurde den Notfallsanitätern durch den Gesetzgeber bei Durchführung von Heilkunde Rechtssicherheit gegeben.

Wenn Notfallsanitäter erlernte und beherrschte Maßnahmen vornehmen, um Lebensgefahr oder wesentliche Folgeschäden für Patienten abzuwenden, müssen sie sich nicht länger ausschließlich auf den rechtfertigenden Notstand berufen [6]. Durch diese Kompetenzerweiterung der Notfallsanitäter ist es beispielsweise nicht mehr zwingend erforderlich, dass Notärzte für *sämtliche* Einsätze, die eine medikamentöse Therapie erfordern, ausrücken: Vielmehr können zumindest manche dieser Einsätze – unter bestimmten Voraussetzungen – nunmehr regelhaft und eigenständig auch von Notfallsanitätern geleistet werden, ohne dass auf diese Weise ein grundsätzlicher bzw. systemischer Verzicht auf die weiterhin wichtige Ressource „Notarzt“ angestrebt wird.

Es wurden aber nicht nur die medizinisch-fachlichen Aspekte in der Ausbildung ausgeweitet. Die entsprechenden Rahmenlehrpläne sehen zudem eine Vielzahl von Unterrichtseinheiten vor, die unter dem Begriff „Management“ subsumiert werden können [7]. Somit ist der Schwerpunkt von der reinen Versorgung von Notfallpatienten hin zu einer ganzheitlich ausgerichteten Berufsausbildung verlagert worden, die nunmehr auch die Bereiche beleuchtet, die für zukünftige Führungskräfte im Rettungsdienst von Bedeutung sind.

### Notärztliche Versorgung

Mit den skizzierten Veränderungen durch die Etablierung des Berufsbildes „Notfallsanitäter“ ergeben sich auch Veränderungen für die Aufgaben und Rolle des Notarztes im Rettungsdienst. Notärzte werden in allen deutschen Rettungsdienstbereichen auf verschiedenen Rettungsmitteln wie Notarzteinsatzfahrzeug (NEF), seltener Notarztwagen (NAW), Intensivtransportwagen (ITW) und Rettungs‑/Intensivtransporthubschraubern (RTH/ITH) eingesetzt. Darüber hinaus sind erfahrene Leitende Notärzte (LNA) für die Bewältigung besonderer Schadensereignisse als Teil einer gemeinschaftlichen Einsatzleitung Rettungsdienst mit Organisatorischen Leitern Rettungsdienst wichtiges Element der Führungs- und Einsatzstrukturen der Gefahrenabwehr und des Bevölkerungsschutzes und tragen im Einsatz die medizinische Gesamtverantwortung.

Die bereits erläuterte Kompetenzerweiterung der Notfallsanitäter soll unter anderem zu einer Entlastung der notärztlichen Einsatzressourcen führen. Auf diese Weise behält der Notarzt seinen Stellenwert in der Notfallversorgung, insbesondere in sehr kritischen und komplexen Notfallsituationen, um dann weitere, ergänzende ärztliche Therapieoptionen bereitzustellen, die von Notfallsanitätern definitiv nicht abgedeckt werden können. Zukünftig wird es daher zu erwarten sein, dass sich die notärztliche Versorgung weiter spezialisiert und einige medizinische Eingriffe, die bisher nur innerklinisch möglich waren, sich zunehmend auch präklinisch realisieren lassen. Experimentelle Versorgungskonzepte wie der Einsatz von speziell ausgestatteten und personell besonders qualifiziert besetzten Notarzteinsatzfahrzeugen (Medical Intervention Car; Heidelberg) oder auch die Verfügbarkeit von Blutprodukten auf bestimmten Luftrettungsmitteln stellen zusätzliche qualitative Herausforderungen für die spezifisch notärztliche Versorgung dar [8]. Mit Einzug von hochinvasiven Therapiekonzepten wie eCPR (ECMO-gestützte Reanimation) oder auch der Clamshell-Thorakotomie ergeben sich darüber hinaus neue Ausbildungs- und Trainingsanforderungen für alle Mitglieder von Rettungsteams.

Auch die Qualifizierung von Notärzten an sich wird in letzter Zeit häufiger diskutiert. Jüngst hat das Land Berlin umfassende Anforderungen an die Qualifizierung von Notärzten, inklusive einer kompetenz- und erfahrungsbasierten Abstufung, etabliert und für die Hauptstadt verbindlich vorgegeben, die weit über die Forderungen der Weiterbildungsordnung hinausgehen und somit einen beispielhaften Qualifizierungsweg aufzeigen [9]. Die zunehmende Verbreitung von telemedizinischen Versorgungskonzepten („Telenotarzt“) führt ebenfalls zu neuen Qualifizierungsbedarfen und Tätigkeitsfeldern für notärztliches Personal im Rettungsdienst [10].

An der Schnittstelle Klinik ist mit der Etablierung der Fachweiterbildung „Klinische Notfall- und Akutmedizin“ eine wichtige Verbesserung sowohl der Zusammenarbeit der unterschiedlichen Professionen als auch der interdisziplinären Patientenversorgung zu erwarten, da erstmalig eine umfassende und weitgehende Zusatzqualifizierung im spezifisch notfallmedizinischen Rahmen eingeführt wurde [11]. Weiterhin steht allerdings die Forderung im Raum, einen „Facharzt für Notfallmedizin“ zu etablieren, der sowohl im klinischen als auch präklinischen Bereich die hohe Bandbreite der Notfallmedizin abbilden kann [12]. Dieser Facharzt sollte über eine inter- beziehungsweise transdisziplinäre Qualifizierung verfügen, um die vielfältigen Schnittstellen und Erfordernisse der alltäglichen und speziellen Notfallversorgung noch besser abdecken zu können als bisher.

Die herausragende Rolle der „Ärztlichen Leiter Rettungsdienst“ (ÄLRD) wurde bundesweit zwar bereits durch die Etablierung des NotSanG im Jahre 2014 gestärkt, allerdings war die Funktion eines ÄLRD zu diesem Zeitpunkt noch gar nicht in allen Bundesländern verbindlich und flächendeckend vorgesehen. In Baden-Württemberg wurde die Funktion formell beispielsweise erst 2018 geschaffen und auch erst im Folgejahr in der rettungsdienstlichen Praxis etabliert [13]. Die ÄLRD verantworten als höchste medizinische Leitungsfunktion die rettungsdienstliche Qualitätssicherung, entwickeln Handlungsleitlinien und gestalten gemeinsam mit den anderen beteiligten Professionen sowie der Verwaltung die Rettungsdienststrukturen. Die konkrete Umsetzung in den Landesrettungsdienstgesetzen, auch mit Blick auf die Vorgaben zu heilkundlichen Maßnahmen von Notfallsanitätern und weitere Standardarbeitsanweisungen, ist allerdings bundesweit heterogen [14]. In der Umsetzung der qualitätssichernden Maßnahmen sind die ÄLRD auf jeden Fall in der zentralen Schlüsselrolle zu sehen; hier sind sie beispielsweise dafür zuständig, die medizinische Supervision der Notfallsanitäter sicherzustellen und dafür geeignete Feedback- und Trainingsinstrumente zu etablieren.

### Gesellschaftliche Rahmenbedingungen

Die Bevölkerungsstruktur der Bundesrepublik Deutschland wird sich in den kommenden Jahren wesentlich verändern. Bereits im mittelfristigen Prognosezeitraum wird eine Abnahme der Erwerbsfähigen bei gleichzeitigem Anstieg der berenteten Menschen erwartet [15]. Für den Rettungsdienst ist dies in mehrfacher Hinsicht relevant: So hat dies zur Folge, dass immer häufiger ältere, multimorbide Menschen mit chronisch degenerativen Erkrankungen zu versorgen sind. Die Stelle zur trägerübergreifenden Qualitätssicherung im Rettungsdienst Baden-Württemberg präsentiert in ihrem jüngsten Bericht Anteile an Notarzteinsätzen von 44 % bei den über 70-Jährigen und weitere 14 % bei den über 60-Jährigen [16]. Es wird erwartet, dass sich dieser Anteil in den nächsten Jahren noch erhöhen wird [17, 18]. Andererseits hat der demografische Wandel auch Einfluss auf die eigenen Personalstrukturen der Rettungsdienstorganisationen. Seit einiger Zeit sind Zeichen eines Fachkräftemangels bei allen Qualifikationsgruppen unübersehbar geworden, nachdem diese bereits vor über 10 Jahren prognostiziert worden sind [19, 20]. Lücken in der Besetzung von Rettungsdienst- und Notarztstandorten werden quer durch die Republik in Fachmedien und der Presse mit zunehmender Häufigkeit thematisiert.

### Einsatzentwicklung, Phänomene „Nicht-Notfälle“ und „Frequent Caller“

Ein zentrales und bisher weitgehend unbeantwortetes Phänomen findet sich ebenfalls in den Einsatzzahlen. Seit mehreren Jahren beklagen die am Rettungsdienst beteiligten Professionen einen zunehmenden Anteil von Einsätzen, bei denen kein notfallmedizinisch relevantes Geschehen im Vordergrund steht, begrifflich teilweise als „Nicht-Notfälle“ betitelt [21, 22]. Einsätze ohne Notarztbeteiligung bzw. ohne das Vorliegen einer kritischen Erkrankung wachsen deutlich stärker an als die Einsätze mit Notarztbeteiligung [1, 17]. Die Ursachen dafür sind multifaktoriell und bisher nicht vollkommen erforscht. Einige Erklärungsansätze liegen in der verringerten Verfügbarkeit oder Kenntnis ambulanter Versorgungsleistungen (hausärztliche Versorgung, kassenärztlicher Notdienst) und einer damit verbundenen Fehlsteuerung der Patienten. Auch psychosoziale Problemstellungen stellen eine häufige Einsatzindikation dar, häufig dann, wenn personale und soziale Ressourcen zur Bewältigung schwieriger Lebenslagen nicht mehr ausreichen oder schlichtweg versagen [23]. Insbesondere im großstädtischen Setting sind auch hohe Fallzahlen sogenannter Frequent Caller festzustellen [24, 25]. Dabei handelt es sich um Patienten, die regelmäßig und sehr häufig den Rettungsdienst in Anspruch nehmen, oft stehen auch hier soziale Indikationen im Raum [26].

Zur Bewältigung dieser Entwicklung haben sich einige Modellprojekte gebildet, mit deren Hilfe neue angepasste rettungsdienstliche Strukturen und Strategien gestaltet werden können. Exemplarisch sind hier der Gemeindenotfallsanitäter (G-NFS), das Projekt zur „Sektorenübergreifenden ambulanten Notfallversorgung“ (SaN) in Hessen oder auch Projekte zur Etablierung von Notfallkrankentransportwagen (N-KTW) oder Rettungseinsatzfahrzeugen (REF) zu nennen. Erste Ergebnisse zum G‑NFS zeigen, dass der Ansatz einer ambulanten Versorgung durch rettungsdienstliches Personal tragfähig ist, Einsätze von Rettungswagen vermeidet und die Notfallversorgung somit insgesamt entlastet [27]. Eine flächendeckende oder gar bundesweite Implementierung ist bisher jedoch in keinem der Projekte zu verzeichnen. Insgesamt zeichnet sich die für das deutsche Rettungsdienstsystem typische Heterogenität ab, die zu sehr unterschiedlichen Verfügbarkeiten und Entwicklungsständen im Bundesgebiet führt.

### Rechtliche und ökonomische Rahmenbedingungen

Die Rahmenbedingungen für die Struktur und Organisation des Rettungsdienstes werden überwiegend auf Grundlage der jeweiligen landesrechtlichen Regelungen festgelegt und sind damit bezogen auf die Bundesrepublik beispielsweise bezüglich geltender Hilfsfristen und ihrer Definition deutlich heterogen [28]. Dies betrifft auch die Finanzierungslogik, die uneinheitlich aufgebaut ist (Konzessions‑, Submissions-, kommunales Modell) und somit auch unterschiedlichen vergaberechtlichen Vorgaben unterworfen ist [29]. Ausschreibungen von Rettungsdienstleistungen und die damit oft verbundene Anwendung der Bereichsausnahme nach Vergaberecht^1^ fordern Träger und Leistungserbringer gleichermaßen und haben relevanten Einfluss auf die Systemgestaltung. Eine qualifizierte Messung der Systemleistung und Versorgungsqualität anhand von einheitlichen Kennzahlen und die Festlegung eines Vergleichsmaßstabs über Ländergrenzen hinweg erfolgen nicht [30]. Eine bundeseinheitliche Strategiebasis, die zugleich Grundlage für eine einheitliche Entwicklung des Rettungsdienstes wäre, ist nicht erkennbar. Experimentierklauseln zur Exploration neuer Strukturen in der Daseinsvorsorge und zur Herstellung gleichwertiger Lebensverhältnisse über einen wirkungsorientierten Ansatz sind in aller Regel nicht Bestandteil der aktuellen Gesetzgebung [31].

Aus dem Spannungsfeld zwischen gesellschaftlichen und strukturellen Anforderungen resultieren für den Rettungsdienst regelmäßig weitere Herausforderungen. Die Einführung der DRG-Fallpauschalen (Diagnosis-related Groups) im Jahre 2003 war z. B. mit erheblichen Auswirkungen auf die Bereiche Sekundäreinsätze und qualifizierter Krankentransport verbunden, weil Patienten erheblich schneller entlassen wurden, um die klinische Bettenauslastung und Verweildauer möglichst kosteneffizient zu gestalten. In einzelnen Rettungsdienstbereichen sind die Einsatzzahlen seinerzeit dadurch um 50 % gestiegen [32]. Auch der Bereich der kassenärztlichen Versorgung sowie der ärztlichen Bereitschaftsdienste entfaltet Effekte auf den Rettungsdienst. Fehlende Kenntnisse der Rufnummer 116 117 bei der Bevölkerung, unzureichende Verfügbarkeiten oder Erreichbarkeiten als auch zu geringe Hausbesuchskapazitäten an sich führen zu einer erhöhten Einsatzbelastung der Rettungsdienste und der Notaufnahmen und damit zu einer zumindest anteilig fehlerhaften Ressourcenbelastung [33].

Die dem Grunde nach wichtige und überfällige Reform der Notfallversorgung in Deutschland wird zwar intensiv diskutiert [34, 35], es fehlt allerdings weiterhin an einem auf breiter politischer und fachlicher Basis getragenen Umsetzungsvorschlag für ein patientenorientiertes und effizientes Notfallmanagement. Der politische Beratungsprozess und der fachliche Diskurs werden aktuell durch Erfahrungen aus der COVID-19-Pandemie mit einer neuen Perspektive angereichert, nämlich der Frage nach den Belastungsgrenzen des derzeitigen Systems [36]. Auch die längst überfälligen Diskussionen rund um die Organisation und die Geschäftsprozesse von Integrierten Leitstellen werden bundesweit durch die jeweiligen Träger mit teilweise sehr unterschiedlicher Intensität und Zielrichtung geführt [37]. Es ist nicht abzusehen, dass die Standards angeglichen werden, wenngleich dies zur Integration digitaler Innovationen wie Smart City (Ausgestaltung urbaner Räume mit intelligenten Technologien) und dem Internet of Things (Verknüpfung von Geräten über das Internet) absehbar erforderlich wäre [38].

### Medizinische und technische Rahmenbedingungen

Das Wissen im Bereich der (notfall-)medizinischen Versorgung nimmt rasant zu und auch die technischen Möglichkeiten entwickeln sich beständig weiter. Heutige Rettungswagen sind mit umfangreicher Medizintechnik ausgestattet. Neue Gerätschaften (z. B. für mechanische Reanimationshilfe (mCPR), Sonografie oder Blutgasanalysen) sind zunehmend verbreitet. Dies führt zu steigenden Anforderungen an das Personal selbst als auch an die technische und administrative Verwaltung. Auch telemedizinische Versorgungskonzepte (z. B. Telenotarzt und/oder Telenotfallmedizin) sind inzwischen in vielen Bereichen etabliert oder ihre Einrichtung ist in Kürze vorgesehen [39]. Zukünftig bleibt noch offen, inwiefern Systeme des Ambient Assisted Living (kurz AAL; Alltagsunterstützung für ältere und andere unterstützungsbedürftige Menschen), Gesundheits-Apps und das Internet of Things in die Prozesse der Gesundheitsversorgung eingebunden werden können und diese beeinflussen.

### Digitalisierung im Rettungsdienst

Nationale Register haben den Rettungsdienst im Hinblick auf die Gestaltung und Nutzung digitaler Prozesse bisher als leistungsschwach (Low-Performer) identifiziert [16, 40]. Mit zunehmender Digitalisierung im Rettungsdienst kann hier entgegengewirkt werden. Wie eine deutschlandweite, explorative Online-Befragung von Notärzten und Rettungsdienstmitarbeitern zeigt, sind einige Innovationen, wie beispielsweise die digitale Einsatzübermittlung auf das Navigationssystem der alarmierten Rettungsmittel, im Bundesgebiet annährend flächendeckend im Einsatz. Anders sieht es hingegen bei der bisherigen Implementierung einer telemedizinischen Voranmeldung von Notfallpatienten sowie einer Übermittlung von Datensätzen wie Bildern, neurologischen Scores oder einem 12-Kanal-Elektrokardiogramm an das Zielkrankenhaus aus. Auch die audiovisuelle Konsultation eines Telenotarztes ist aktuell in den wenigsten Regionen möglich [41].

Ein flächendeckender Einsatz der genannten Informations- und Kommunikationstechnologien (IKT) ist zeitnah anzustreben. So konnten langjährige Projekte, wie beispielsweise das „Stroke-Angel“-Projekt, bereits aufzeigen, dass der Einsatz von telemedizinischen Anwendungen zu einer verbesserten Patientenversorgung führen kann [42]. Die Etablierung solcher IKT liegt in der Verantwortung der Managementebene und ist mitunter nicht nur kosten- und zeitintensiv in der Umsetzung, sondern bedarf auch unterschiedlicher Einweisungs- und Schulungsangebote für die Mitarbeiter. Nur mit Hilfe durchdachter Umsetzungskonzepte können neue Technologien mittel- bis langfristig Bestand haben und gleichzeitig die steigenden Anforderungen der Mitarbeiter berücksichtigen [43].

### Bedeutung von besonderen Gefahrenlagen

Nicht zuletzt unter dem Eindruck des Krieges in der Ukraine und den Hochwasserereignissen in Rheinland-Pfalz und Nordrhein-Westfalen ist auch wieder eine zunehmende Präsenz von Themen der Krisenvorsorge und -vorbereitung im Kontext des Bevölkerungsschutzes zu beobachten [44]. Es steht zu erwarten, dass die Notfallversorgung im Lichte dieser Herausforderungen einer Untersuchung hinsichtlich ihrer Resilienz, der Doppelverplanung von Ressourcen und der Reservenbildung unterzogen werden muss. Darüber hinaus sind zunehmende Vulnerabilitäten kritischer Infrastrukturen (KRITIS), wie z. B. der Energieversorgung und der Kommunikation, zu betrachten.

Die Pandemie hat den gesamten Gesundheitsbereich vor immense Herausforderungen gestellt und einige Schwächen sichtbar gemacht [36]. Insbesondere die Abhängigkeit von Lieferwegen ist zu Beginn der Pandemie deutlich geworden. Zeitweise verfügten einzelne Rettungsdienstbereiche nur noch über Infektionsschutzmaterial für wenige Tage [45]. Einheitliche rechtliche Vorgaben oder Empfehlungen zur Bevorratung gibt es bisher nicht bzw. vorhandene Vorgaben müssen an die Erfahrungen der Pandemie angepasst werden [46]. Die Betrachtung von Risiken ist für zukünftige Ressourcen- und Einsatzplanungen essenziell. Der Rettungsdienst, als Element der umfassenden Gesundheitsversorgung in Deutschland, zählt zweifelsfrei zu den KRITIS. Die planerische Vorbereitung auf unterschiedlichste Szenarien, handfeste Risikoanalysen auf allen Organisationsebenen und krisenfeste trainierte Strukturen sind Management- und Systemaufgabe. Auch Facetten wie die Bevorratung und das Supply-Chain-(Continuity‑)Management sind essenziell, um einen resilienten handlungsfähigen Rettungsdienst aufzustellen [47]. Dies muss auch in den jeweiligen Rettungsdienstgesetzen verankert werden, um die notwendigen Ressourcen und Finanzierungen sicherzustellen.

## Anforderungen an das Rettungsdienstmanagement

Vor dem Hintergrund der bisherigen Ausführungen zeichnet sich ab, dass bisher übliche, historisch gewachsene „Managementlösungen“ aus den Anfangszeiten der Notfallrettung in den 1970er- und 1980er-Jahren nicht länger zielführend sein können [48]. Tradierte Abläufe und Strukturen funktionieren nicht mehr. Erhebungen über die Qualifikation von Führungs- und Leitungskräften im Rettungsdienst gibt es nicht. Insbesondere in den unteren Führungsebenen ist jedoch anzunehmen, dass der Großteil der aktiven Führungskräfte keine entsprechende Weiterbildung absolviert hat, bevor die Funktion übernommen wurde. Ohne das Engagement und die Leistungen Einzelner an dieser Stelle pauschal in Abrede stellen zu wollen, kann man dies nur als suboptimal bezeichnen. Allerdings sind keinesfalls diejenigen zu kritisieren, die aus verständlichen Gründen und höchst nachvollziehbar eine für sie günstige Aufstiegschance genutzt haben, sondern v. a. diejenigen, die für derart semiprofessionelle Rahmenbedingungen innerhalb des Rettungsdienstes verantwortlich sind. Um zukünftigen Anforderungen gerecht zu werden, ist eine Professionalisierung des Rettungsdienstmanagements nicht nur wünschenswert, sondern dringend geboten [49].

Diese Auffassung wird im Übrigen auch aus der Praxis bestätigt: So sind 96 % der in einer Studie befragten Rettungswachenleiter selbst der Meinung, dass ein Bedarf an adäquaten Weiterbildungsmöglichkeiten besteht [50]. Auch die Auseinandersetzung mit komplexen systemischen, gesellschaftlichen und politischen Fragen und die Konzeption von Antworten wird als strategische Managementaufgabe erfordern, dass zukünftige Leitungsfunktionen eine fundierte wissenschaftliche Qualifizierung erhalten. Der tatsächliche Bedarf daran lässt sich mit Blick auf das zunehmende Studienangebot bestätigen. Ergänzt werden diese Rollen durch die Ärztlichen Leitungen Rettungsdienst (ÄLRD); gemeinsam entsteht ein interdisziplinäres Rettungsdienstmanagement, das die sich verändernden Strukturen aktiv gestaltet.

### Personalmanagement

Als wesentlichste Herausforderungen an das Rettungsdienstmanagement dürften eindeutig alle personalbezogenen Themen zu betrachten sein. Bereits heute gibt es deutliche Anzeichen für einen Fachkräftemangel im Gesundheitswesen, von dem auch der Rettungsdienst betroffen ist [20]. Die vorgestellte Problematik verstärkter Vorhaltungen zur Bewältigung des Einsatzaufkommens verschärft den Fachkräftemangel zusehends. Verweilzeiten im Beruf, Wechsel aus dem Rettungsdienst in andere Bereiche oder Branchen sind nach wie vor problematisch zu werten. Der War for Talents, d. h. der Kampf um qualifizierte Fachkräfte, ist somit auch im Rettungsdienst angekommen und betrifft de facto alle Qualifikationsstufen und Berufsgruppen.

Während die Rekrutierung von Auszubildenden bisher keine Schwierigkeiten bereitet, stellt die Bindung der Mitarbeiter eine immense Herausforderung dar. Etwa die Hälfte der Absolventen plant eine Verweildauer im Beruf von weniger als 10 Jahren [51].

Ein wesentliches Defizit liegt in den bisher fehlenden oder nur gering ausgeprägten Weiterbildungsmöglichkeiten im Rettungsdienst. Gängige Zusatzqualifikationen (beispielsweise Praxisanleiter oder Desinfektor) ermöglichen kein wirkliches berufliches Vorankommen. Mit zunehmender Akademisierung und neuen Studiengängen für Rettungsdienstmitarbeitende können demgegenüber durchaus interessante Optionen verbunden sein. Auch die Tätigkeiten als Gemeindenotfallsanitäter Field Supervisor (z. B. Wien) sowie Rotationsmodelle in zentralen Notaufnahmen bieten Möglichkeiten einer Job Rotation (Wahrnehmung verschiedener Funktionsstellen im Wechsel) oder eines Job Enrichment (qualitative Aufgabenerweiterung). Selbstverständlich sind zukünftig auch Entwicklungsmaßnahmen in Richtung der administrativen Bereiche oder ins Management auf allen Ebenen möglich und zukunftsweisend. Arbeitgeberattraktivität und -bindung werden maßgebliche Wettbewerbsfaktoren sein, die langfristig und strategisch zu planen sind, um leistungs- und wettbewerbsfähig zu bleiben.

Mit Blick auf das im Rettungsdienst und in der Notfallmedizin ständig erforderliche Lernen fällt auch der Gestaltung von Fortbildungsmaßnahmen eine wichtige Rolle zu. Es kann postuliert werden, dass die derzeitigen gesetzlichen Fortbildungsverpflichtungen von etwa 24–38 h (vgl. Bayern und Hessen) unzureichend sind, um auf die vielfältigen Neuerungen und vor allem Trainingsbedarfe der Notfallmedizin eingehen zu können. In den nächsten Jahren ist zu erwarten, dass hochrealistische Simulationsangebote die Aus- und Fortbildung auch qualitativ wesentlich anheben werden. Idealtypisch werden diese Angebote interdisziplinär mit dem rettungsdienstlichen und ärztlichen Personal gemeinsam angeboten und selbstverständlich sein.

Grundlage für eine bessere Qualifizierung und Fortentwicklung der Angebote sind die ebenfalls verbesserte Qualifikation der Praxisanleitenden und die inzwischen zunehmend akademisch-pädagogischen Qualifikationen der Lehrkräfte an Rettungsdienstschulen. Auch für Beschäftigte in integrierten Leitstellen sind neue Qualifizierungsmodelle zu schaffen, die sowohl eine modulare Weiterqualifizierung aus dem Einsatzdienst heraus zulassen, diese aber nicht zur zwingenden Voraussetzung machen, als auch den Gedanken eines eigenständigen Berufsbildes aufgreifen [52].

### Gesundheitsmanagement und Führungsverhalten

Mit Blick auf die Lebensarbeitszeit ist es erforderlich, betriebliche Gesundheitsmanagementsysteme einzurichten, um die Gesundheit und Leistungsfähigkeit der Mitarbeiter langfristig erhalten zu können. In den letzten Jahren wurde ein nahezu flächendeckendes psychosoziales Unterstützungsangebot etabliert, das Fehlbelastungen und Fehlbelastungsfolgen nach Extremsituationen entgegenwirkt [53]. Einsatzbezogene Belastungen stellen allerdings nur eine Facette der möglichen Belastungsfaktoren für Einsatzkräfte dar. Der Alltag im Einsatzdienst und auf der Rettungswache bietet zahlreiche weitere mögliche Belastungen, die unterschiedlich wahrgenommen und verarbeitet werden [54]. Die Gestaltungsspielräume der Organisationen und Verantwortlichen bieten Möglichkeiten, Belastungsfaktoren zu verringern, ein ansprechendes und angenehmes Arbeitsumfeld zu schaffen und somit Attraktivitätsfaktoren zu schaffen.

Darüber hinaus kommt der Arbeitsplatzgestaltung und den Partizipationsmöglichkeiten für Mitarbeiter eine größere Bedeutung zu [54]. Hier sind Veränderungen im Führungsstil und -verständnis angebracht. Partizipative Ansätze und eine offene, gesundheitsfördernde Kultur mit gemeinschaftlichen Werten wirken sich nachweislich positiv auf die Gesundheit, Stimmung und Motivation der Beschäftigten aus [55]. Nicht zuletzt müssen auch neue Modelle zur Arbeitszeitgestaltung geschaffen werden, um unterschiedliche Anforderungen an die Work-Life-Balance, die berufliche und akademische Weiterbildung und die Bedürfnisse unterschiedlicher Lebensabschnitte zu adressieren.

## Wissenschaft im Rettungsdienst

Ein wesentliches Defizit in der deutschen Rettungsdienstlandschaft sind die bisher nur schwach ausgeprägten Forschungsaktivitäten. Das spezifische wissenschaftliche Fundament durch empirische Studien nimmt zwar in den letzten Jahren kontinuierlich zu, fehlt aber in vielerlei Hinsicht weiterhin. Tatsächliche Vor- und Nachteile verschiedener Organisations- und Finanzierungsformen rettungsdienstlicher Leistungen sind beispielsweise unklar; das System des deutschen Rettungsdienstes wird – gerade aufgrund seiner aktuellen, fast unüberschaubaren Heterogenität – sehr zu Recht als Blackbox bezeichnet [29]. Standardisierte und nutzbar aufbereitete Datenquellen und entsprechende Analysen sind eine Seltenheit. Die Statistiken der Länder sind uneinheitlich aufbereitet und basieren auf unterschiedlichen strukturellen Vorgaben, so dass eine Vergleichbarkeit nur bedingt gegeben ist.

Während in anderen medizinischen Bereichen eine Vielzahl wissenschaftlicher Fachgesellschaften zu verzeichnen ist, steht der Rettungsdienst hier noch am Anfang. Erst in den letzten Jahren sind mit der Deutschen Gesellschaft für Rettungs- und präklinische Notfallmedizin sowie der Deutschen Gesellschaft für Rettungswissenschaften Verbände gegründet worden, die sich eingehend mit rettungsdienstlichen Fragestellungen befassen.

Studiengänge mit rettungsdienstlichem (Management‑)Schwerpunkt gab es bis vor wenigen Jahren faktisch nicht. Inzwischen bestehen hier bundesweit unterschiedliche Ansätze und Angebote, die auch zunehmend Interessenten finden und eigene Disziplinen mit feldspezifischer Forschung aufbauen.

## Organisatorische und strukturelle Konsequenzen

Das bekannte Modell der Rettungskette nach Ahnefeld [3], mit dem der Ablauf der einzelnen Hilfsleistungsschritte nach einem Notfall idealisiert veranschaulicht wird, erscheint in seiner sich weiterentwickelnden und ausdifferenzierten Umwelt nicht mehr in vollem Umfang zeitgemäß, zumal es sich ausschließlich auf den medizinischen Aspekt rettungsdienstlicher Leistung, nicht aber auf die Einbettung des Rettungsdienstes in das Gesamtsystem der Gesundheits- bzw. Notfallversorgung bezieht. Hier sind in den kommenden Jahren alternative, deutlich umfassendere Organisationsmodelle zu entwickeln. Vor allem werden sich Rettungsdienste stärker mit anderen Akteuren der Notfallversorgung vernetzen müssen als bisher, insbesondere auch mit den Anbietern der psychosozialen Akutversorgung, aber gerade auch mit dem ärztlichen Bereitschaftsdienst [21]. Leitstellen müssen sich als digital hoch vernetzte, barrierefreie und dienstleistungsorientierte Einrichtungen mit einem hohen Grad an Standardisierung ihrer Leistungen für die Daseinsvorsorge und Gefahrenabwehr herausbilden [56]. Sowohl für Rettungsdienste als auch für integrierte Leitstellen dürften sich die besonderen Herausforderungen der Professionalisierung, der Standardisierung, aber auch im Personalmanagement nur durch größere Organisationsstrukturen wirtschaftlich bewältigen lassen.

Kostendruck und Engpässe bei der Gewinnung von Fachkräften werden es erforderlich machen, die Vorhaltung rettungsdienstlicher Ressourcen kritisch zu überprüfen und ggf. zu optimieren. Insbesondere in ruralen Bereichen wird dabei ein Abwägen zwischen betriebswirtschaftlichen, medizinischen und ethischen Überlegungen unvermeidlich sein. In diesem Kontext ist nicht zuletzt z. B. auch auf bereits entwickelte Konzepte von temporären Rettungswachen und die Einbindung von Spontanhelfenden in der Nähe von Ereignisorten (sogenannte App-Retter) hinzuweisen.

Betriebswirtschaftlich optimiertes Management zur Effektivitätssteigerung des Rettungsdienstes hat gleichermaßen seinen Platz wie fachlich getragene Führung mit dem Schwerpunkt organisatorischer und strategischer Weiterentwicklung und Mitarbeiterbindung. Ein ausgewogenes Angebots- bzw. Dienstleistungsprofil hilft, sich von Mitbewerbern abzugrenzen und zugleich ein wirkungsorientiertes Netzwerk zu unterhalten. In diesem Bereich liegen vielfältige Herausforderungen für ein künftiges Rettungsdienstmanagement, welches auf den verschiedensten Organisationsebenen eine wissenschaftliche Ausbildung erfordert und im Zusammenwirken mit ärztlicher Kompetenz evidenzbasiert wirkt.

## Ausblick

Spätestens mit der Einführung des Berufsbildes Notfallsanitäter hat eine umfassende Professionalisierung des Rettungsdienstes von einer medizinischen Transportdienstleistung hin zu einer umfassenden Gesundheitsdienstleistung eingesetzt. Der Rettungsdienst ist dabei gleichermaßen das Spiegelbild von gesellschaftlichen Notwendigkeiten und ökonomisch realisierbaren Möglichkeiten. Derzeit stellt der Rettungsdienst die einzige mobile Hilfsinstanz dar, die rund um die Uhr medizinische Notrufe bedienen kann und muss. Dabei kommt der Rettungsdienst auch häufig bei sozialen oder psychiatrischen Situationen zum Einsatz, die nicht unbedingt zum ursprünglichen Aufgabenportfolio gehören. Für diese Entwicklungen stehen erste Lösungsansätze zur Verfügung, die allerdings noch flächendeckend evaluiert und bei Eignung zur Anwendung gebracht werden müssen. Die Überwindung sektoraler Grenzen wird dabei ebenso eine Rolle spielen wie die Harmonisierung der rettungsdienstlichen Strukturen auf Länder- und Bundesebene. Die Träger und Leistungserbringer sind gleichermaßen gefordert innovative Lösungsansätze zu generieren, zu evaluieren und umzusetzen.

Im operativen und strategischen Management wird es erforderlich sein, attraktive und mitarbeiterorientierte Rahmenbedingungen zu schaffen, welche eine langfristige Personalentwicklung und somit Bindung ermöglichen. Es gilt die berufliche Entwicklungsperspektive zu gestalten und die bisherige Lücke an echten Weiterbildungsmöglichkeiten aufzufüllen.

Die Defizite an empirischer Forschung sind mit Blick auf die vorgestellten Herausforderungen und zukünftigen Anforderungen aufzulösen. Aktuelle Fragen und Diskussionen der Versorgungs- und Organisationsstrukturen im Rettungsdienst machen eine valide Erarbeitung von Antworten zwingend erforderlich, um tragfähige, effektive und nachhaltige Lösungen zu schaffen. Die zunehmende Akademisierung sowohl der Führungs- und Leitungskräfte, der Dozenten als auch der Notfallsanitäter bietet dabei die wesentliche Chance zur Bereitstellung der Kompetenzen, die zur Systemgestaltung notwendig sind. Gleichberechtigt sind die Strukturen der Ärztlichen Leitungen Rettungsdienst interdisziplinär und kooperativ im Gesamtmanagement zu verankern, um die Fragen medizinischer Qualitätssicherung und Systementwicklung gebührend zu tragen und zu verantworten.

Mit Blick auf die aktuellen Reformvorhaben im Gesamtsystem der Gesundheitsversorgung ist zu postulieren, dass die Entwicklung und Gestaltung zukunftsfähiger Strukturen nur gelingen werden, wenn alle Akteure einen offenen und konstruktiven Austausch leben und gemeinsam agieren. Es gilt also, Systemgrenzen zu überwinden und ein patientenzentriertes System zu schaffen, in dem alle Akteure Hand in Hand arbeiten.

## Footnotes

1 Ausnahmevorschrift für den Rettungsdienst gemäß § 107 Abs. 1 Nr. 4 Gesetz gegen Wettbewerbsbeschränkungen (GWB), nach der eine begrenzte Möglichkeit besteht, das Vergaberecht bei der Vergabe von Rettungsdienstleistungen nicht anzuwenden.
